# The Origin and Evolution of the 18th Century Hospital Movement
*Previous articles appeared on December 13, 1913; January 17 and 31, 1914.


**Published:** 1914-02-14

**Authors:** 


### IV. THE ORIGIN AND EVOLUTION OF THE 18th CENTURY HOSPITAL MOVEMENT.*

The Westminster Infirmary.

The choice of premises fell upon a house in Broad Sanctuary, but the owner, on learning the uses to which it was intended that the property should be employed, cancelled the lease; and eventually the infirmary was established in Petty France, and the first in-patient was admitted May 11, 1720. Considering the limitation of choice to St. Margaret's parish, the selection of a site adjoining Tothill Fields was likely enough a wise one, though the low level of the land south of Piccadilly, the mists hovering over the canal and marshes of St. James's Park, and the vapours arising from the river in the further distance, then as now, were not the best environment for a hospital. Such, indeed, was the argument voiced thirteen years later, when it became necessary to vacate the premises in Chapel Street (hard by) to which the infirmary had been removed in 1724 by those of the Westminster subscribers who eventually seceded to the more salubrious situation at Hyde Park Corner, where they established St. George's Hospital. The advantages of sanitary surroundings were then so little appreciated that, as has been seen, Poor-Law infirmaries were not infrequently erected in contiguity with graveyards and pest-fields. It was another instance of the protection of the healthy without regard to the welfare of the sick.

At their best the premises must have been far from commodious. The wards must have been small, the ceilings low, the passages narrow, and the ventilation deficient, for windows were rarely practicable except in the lower sash. The picture presented to us by similar institutions of a slightly later date leads to the assumption that at first no bathing accommodation whatever existed. One scarcely realises how modern an invention a bathroom is in private dwellings. The floors, some of which, with the passages, were sanded, were washed at intervals, and occasionally sprinkled with vinegar. From time to time the beds were searched for vermin; the wards and bedding were fumigated with wormwood; chafing-dishes and heaters were provided for the purpose of deodorisation; and it was not uncommon for nosegays to be ordered by the surgeon "to prevent the patient from being infected by the stench of his wounds."

The wooden four-post tester bedsteads were provided with curtains to exclude draughts in the winter months, and under each bed was a box or drawer to contain the patient's foul linen. The mattresses were mostly of straw. Coal, and sometimes wood, was burned to warm the wards, and tallow candles gave an uncertain light in the dark hours. The ordinary diet consisted of a pint of milk pottage for breakfast; half a pound of boiled meat on four days of the week, and a pint of pease pottage on the others for dinner; and cheese or broth for supper. Fourteen ounces of bread and a quart of small-beer were allowed daily to each patient, but vegetables are not mentioned.

The sexes were placed in different wards, but medical and surgical cases of every description lay side by side, and it was not always that each patient had a separate bed. The nursing was of a primitive character, the nurses being mostly widows, more or less addicted to drink, while the night nurses, then called "watches," consisted of those who were qualifying for the posts occupied by their more experienced seniors. The choice of the less competent for the more responsible duties of the night is explained by the necessity for the attendance of the most skilful to take the instructions of the staff and assist in the dressing.

Each of the physicians attended at least once a week to admit and discharge patients, and to visit the wards; and the surgeon-in-ordinary every morning to dress his cases and perform minor operations. They attended at other times if required, but neither physician nor surgeon had power to admit cases upon his own authority, except in urgency, that being reserved for the board.

It is somewhat remarkable that the proposal to erect an infirmary at Westminster should have excited so little attention as to be barely mentioned in the contemporary press. But the movement was as yet limited to a small band of philanthropists—the few amongst the many; in an age remarkable for its apathy the general public had not yet been educated up to a realisation of its duty towards its poorer brethren.

The voluntary hospitals differed from the chartered institutions, inasmuch as—

1. They were unendowed and entirely dependent upon the voluntary subscriptions and benefactions of the public.
2. Their administration was in the hands of the subscribers instead of a president and governors appointed by charter.
3. Their medical and surgical staff was honorary, and as subscribers enjoyed the right of voting upon the general management.
4. No fees were required from applicants for admission.

They are said to have formed a midway refuge between the chartered hospitals and the parish workhouse.

"A great multitude of the most miserable persons who were unable to procure the fees necessary for admission to the former, yet whose position did not entitle them to parish relief, must have perished had not the subscription hospitals received them free of all expense," † which statement is corroborated by the following letter from General Oglethorp to Oliver Goldsmith:—

"Cranhal Hall, Essex (n.d.).

"How just, Sir, were your observations that the poorest objects were by extreme poverty deprived of the benefit of hospitals erected for the relief of the poorest. Extreme poverty, which should be the strongest recommendation, is here the insurmountable obstacle which leaves the distressed to perish. The qualifying such objects to receive the benefits of hospitals answered the intentions of the intended society. The design is the immediate relief from perishing, whereby giving time and protection to get proper destinations. And this, of being admitted into a hospital, is a proper destination. You were so good as to offer to distribute such sums as should be sent to you. At the same time that I am to return you thanks for your charitable offer, I am to send you five pounds to be distributed for that purpose in the time and manner you think proper. Which I accordingly herewith send.

"I am, Sir, your obedient humble servant,

"(Signed) J. Oglethorp."

* Previous articles appeared on December 13, 1913; January 17 and 31, 1914.

† Wilson, *Hist. Middlesex Hosp.*, pp. 41, 44.

This letter, written in or after the year 1768, when Oglethorp first made Goldsmith's acquaintance, refers to the fees demanded at the chartered hospitals, and informs us of a charitable society in which the poet was interested. While Oglethorp's efforts on behalf of poor prisoners are well known, this is the sole surviving letter of those which passed between him and Goldsmith, and comprises all the evidence at hand of the latter's charitable connection with hospitals. No stronger condemnation of the exaction of these fees could well be written, and nothing could better describe the resulting situation.

Twenty years later John Howard wrote: "The securities and fees required at admission into many of the hospitals bear hard upon the poor, and absolutely exclude many who have the greatest need of relief. . . . I saw a woman bring her child, and with tears leave the fee of 2s. 9d. for the nurse and 6d. for the steward." *

* Howard, *Account of Prisons, etc.*, 1789.

